# A Fatal Case of Fulminant Panton-Valentine Leukocidin-Positive Methicillin-Resistant Staphylococcus aureus Necrotising Pneumonia in a Previously Healthy Adolescent: A Case Report

**DOI:** 10.7759/cureus.108467

**Published:** 2026-05-08

**Authors:** Zakariya Hogsade, Alex Raggett, Ayodeji Dosu, Maya De Silva-Morgan

**Affiliations:** 1 Critical Care, Sandwell and West Birmingham Hospitals NHS Trust, Birmingham, GBR; 2 Acute Medicine, Sandwell and West Birmingham Hospitals NHS Trust, Birmingham, GBR; 3 Critical Care, The Royal Wolverhampton NHS Trust, Birmingham, GBR

**Keywords:** medical intensive care unit (micu), panton-valentine leukocidin (pvl), pediatric emergencies and critical care, septic emboli, septic shock in children

## Abstract

Panton-Valentine leukocidin (PVL)-producing *Staphylococcus aureus* can cause severe necrotising pneumonia in previously healthy individuals. We report the case of a 17-year-old previously well adolescent who presented with a short prodrome of influenza-like symptoms and rapidly deteriorated into severe respiratory failure and septic shock. Despite early escalation of care, broad-spectrum antimicrobial therapy, and intensive supportive management, the patient developed bilateral pneumothoraces, refractory hypoxaemia, and multiorgan dysfunction. Blood cultures and respiratory samples confirmed methicillin-resistant *Staphylococcus aureus*, with subsequent confirmation of PVL production. This case demonstrates a rapidly progressive clinical course with severe complications and a fatal outcome despite intensive care management.

## Introduction

Panton-Valentine leukocidin (PVL) is a cytotoxin produced by certain strains of *Staphylococcus aureus* and is associated with severe skin and soft-tissue infections and fulminant necrotising pneumonia [1]. PVL exerts its pathogenic effect by forming pores in leukocytes, leading to cell lysis and the release of pro-inflammatory mediators, which contribute to tissue necrosis and pulmonary destruction [2]. PVL-positive pneumonia predominantly affects young, immunocompetent individuals and is characterised by rapid clinical deterioration, haemoptysis, leukopenia, and extensive pulmonary necrosis, with mortality rates that may exceed 50% [1,3]. Early diagnosis is challenging due to its initial similarity to community-acquired pneumonia; however, delayed recognition can be fatal [4]. We present a fatal case of confirmed PVL-positive methicillin-resistant *Staphylococcus aureus *(MRSA) necrotising pneumonia in a previously healthy adolescent to highlight key diagnostic and management considerations.

## Case presentation

A 17-year-old adolescent with no significant past medical history presented with a two-day history of illness. The initial symptoms included myalgia, fever, lethargy, and anorexia, followed by vomiting and diarrhoea. Over the subsequent 24 hours before admission, the patient developed headache, neck pain, and progressively worsening shortness of breath. These symptoms were accompanied by a productive cough with blood-stained, rusty sputum and the appearance of a generalised wheal-like rash. There was no history of recent travel, alcohol consumption, or illicit drug use. The patient reported occasional vaping, and there was no history of exposure to sick contacts.

On admission, the patient was tachypnoeic with a respiratory rate of 31 breaths per minute and hypoxic, with oxygen saturations of 93% on room air, requiring low-flow supplemental oxygen. Chest auscultation revealed bilateral scattered crepitations.

Arterial blood gas analysis performed while receiving 1 L/minute oxygen via a nasal cannula demonstrated hypoxaemia consistent with type 1 respiratory failure, along with metabolic acidosis. Severe hyponatraemia was also noted. Inflammatory markers showed neutrophilia with an elevated C-reactive protein, while coagulation studies revealed a prolonged prothrombin time and elevated international normalised ratio (Table 1). These findings were consistent with significant systemic inflammation, coagulopathy, and metabolic derangement in the context of evolving sepsis.

**Table 1:** Laboratory and arterial blood gas findings on admission.

Parameter	Result	Reference range
Arterial blood gas		
PaO₂ (kPa)	7.06	10–13
PaCO₂ (kPa)	4.3	4.7–6.0
pH	7.315	7.35–7.45
Lactate (mmol/L)	5.17	0.0-1.79
Bicarbonate (mmol/L)	16	22–26
Base excess (mmol/L)	−9.0	−2 to +2
Electrolytes		
Sodium (mmol/L)	118	135–145
C-reactive protein (mg/L)	276	0-5
Haematology		
White cell count (×10⁹/L)	9.2	4.0–11.0
Neutrophils (×10⁹/L)	8.56	2.0–7.5
Lymphocyte (×10⁹/L)	0.22	1.0–4.0
Coagulation		
Prothrombin time (seconds)	24	11–13.5
INR	2.2	0.8–1.2

**Figure 1:**
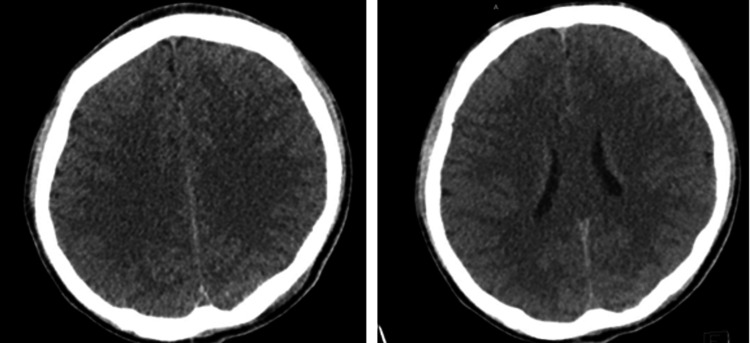
Computed tomography (CT) of the head. Initial CT of the head with representative slices showing no obvious abnormality.

A computed tomography (CT) scan of the head showed no acute intracranial pathology (Figure 1).

Despite initial management, the patient deteriorated within the first 24 hours of admission, with increasing work of breathing and worsening gas exchange. While receiving high-flow nasal oxygen (FiO₂ 0.55 at 50 L/minute), the patient remained tachypnoeic with oxygen saturations of 95%; however, arterial blood gas analysis demonstrated rising carbon dioxide levels and worsening respiratory acidosis (Table 2), consistent with evolving type 2 respiratory failure. The patient also reported increasing fatigue.

**Table 2:** Arterial blood gas analysis demonstrating hypercapnic respiratory failure before intubation.

Parameter	Result	Reference range
PaO₂ (kPa)	10.27	10–13
PaCO₂ (kPa)	7.57	4.7–6.0
pH	7.271	7.35–7.45
Lactate (mmol/L)	1.47	0.0–1.79
Bicarbonate (mmol/L)	22.7	22–26
Base excess (mmol/L)	−2	−2 to +2

Escalation to non-invasive ventilation in the form of bilevel positive airway pressure was unsuccessful, necessitating endotracheal intubation and invasive mechanical ventilation.

**Figure 2:**
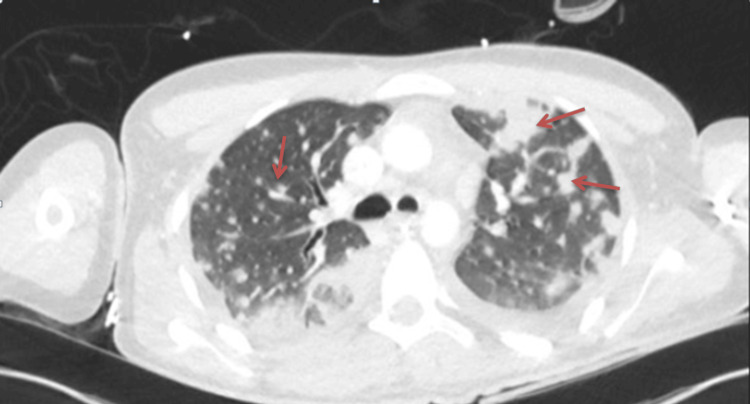
Computed tomography (CT) of the chest. CT of the chest demonstrating bilateral cavitating consolidation and septic emboli (red arrows).

**Figure 3:**
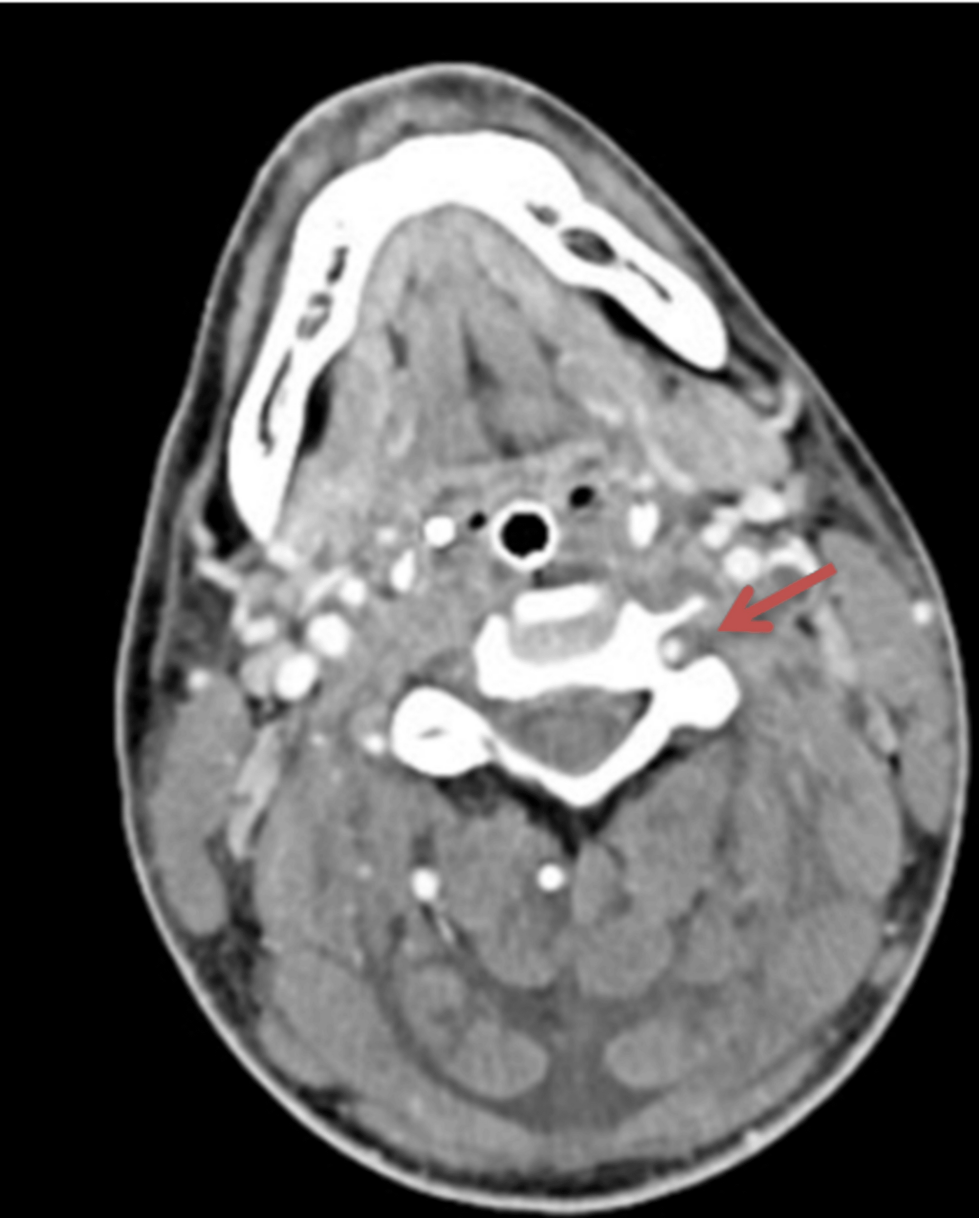
Computed tomography (CT) of the neck. CT of the neck showing a non-occlusive 30-mm-long thrombus in the left internal jugular vein (red arrow).

On day two of admission, following overnight clinical deterioration requiring escalation of respiratory support, CT imaging of the neck, chest, and abdomen revealed extensive bilateral cavitating pulmonary consolidation consistent with necrotising pneumonia and septic emboli (Figure 2), alongside a non-occlusive thrombus of the left internal jugular vein (Figure 3).

Blood cultures grew *Staphylococcus aureus*, subsequently identified as MRSA. MRSA was also isolated from sputum and bronchoalveolar lavage samples. Given the rapid clinical deterioration, haemoptysis, and cavitating changes on imaging in a young patient, PVL-associated infection was suspected and later confirmed by molecular testing.

Differential diagnoses considered included severe community-acquired pneumonia of alternative bacterial or viral aetiology, pulmonary embolism with infarction, and vasculitic processes; however, the clinical and radiological features were most consistent with PVL-associated necrotising pneumonia.

Despite escalation of antimicrobial therapy in consultation with microbiology, including linezolid, clindamycin, rifampicin, and intravenous immunoglobulin, the patient developed refractory hypoxaemia and septic shock requiring vasopressor support. Linezolid and clindamycin were selected for their additional role in suppressing toxin production in PVL-associated infections, while intravenous immunoglobulin was administered as adjunctive therapy to neutralise circulating toxin [5]. The intensive care course was complicated by bilateral tension pneumothoraces requiring multiple intercostal chest drains, persistent air leaks, and surgical emphysema.

Transthoracic and transoesophageal echocardiography showed no evidence of infective endocarditis. The patient was referred to a regional extracorporeal membrane oxygenation service due to refractory hypoxaemia despite maximal ventilatory support. However, the referral was subsequently closed following transient clinical improvement, which was not sustained.

Despite maximal supportive therapy, the patient suffered sudden cardiorespiratory collapse with hypoxia followed by hypotension and pulseless electrical activity. Resuscitative efforts were unsuccessful, and the patient died within 14 days of admission.

## Discussion

PVL-positive MRSA necrotising pneumonia is associated with rapid lung parenchymal destruction, alveolar haemorrhage, and an exaggerated inflammatory response, leading to high rates of respiratory failure and mortality [1,3]. The toxin induces leukocyte lysis and promotes the release of pro-inflammatory cytokines, contributing to extensive tissue necrosis and systemic toxicity [2]. Hallmark clinical features include haemoptysis, cavitating pneumonia, leukopenia, and severe hypoxaemia, all of which were observed in this case.

PVL-associated pneumonia frequently follows a viral prodrome, particularly influenza, which may facilitate bacterial invasion and contribute to the fulminant disease course [6]. Therefore, early clinical suspicion is critical, especially in young, previously healthy individuals presenting with rapidly progressive community-acquired pneumonia and haemoptysis. Prompt initiation of antimicrobial therapy with agents that inhibit toxin production, such as clindamycin and linezolid, is recommended. Adjunctive therapies, including intravenous immunoglobulin, have been used in severe cases to neutralise circulating toxin, although robust evidence from randomised controlled trials remains limited [7].

The development of bilateral pneumothoraces in this patient reflects extensive pulmonary necrosis and alveolar disruption, representing a recognised but severe complication that significantly complicates ventilatory management [8]. Despite advances in intensive care, including consideration of ECMO for refractory hypoxaemia, outcomes remain poor in patients with fulminant PVL-positive MRSA pneumonia.

This case reinforces the importance of early recognition, aggressive antimicrobial therapy, and multidisciplinary management. However, it also highlights the persistently high mortality associated with this condition, even in advanced intensive care settings.

## Conclusions

PVL-positive MRSA necrotising pneumonia is a rare but highly fatal condition that predominantly affects young, otherwise healthy individuals and is characterised by a rapidly progressive clinical course. This case highlights the importance of maintaining a high index of suspicion in patients presenting with severe community-acquired pneumonia, particularly when associated with haemoptysis, hypoxaemia, and cavitating lung changes. Early recognition, prompt microbiological diagnosis, and initiation of toxin-suppressing antimicrobial therapy are critical to management. However, despite aggressive supportive care and advanced intensive care interventions, including escalation of respiratory support, outcomes remain poor. This case underscores the need for heightened clinical awareness and early multidisciplinary involvement when managing suspected cases of PVL-associated necrotising pneumonia.
